# Influence of Tumor Microenvironment and Fibroblast Population Plasticity on Melanoma Growth, Therapy Resistance and Immunoescape

**DOI:** 10.3390/ijms22105283

**Published:** 2021-05-17

**Authors:** Veronica Romano, Immacolata Belviso, Alessandro Venuta, Maria Rosaria Ruocco, Stefania Masone, Federica Aliotta, Giuseppe Fiume, Stefania Montagnani, Angelica Avagliano, Alessandro Arcucci

**Affiliations:** 1 Department of Public Health, University of Napoli “Federico II”, 80131 Naples, Italy; veronica.romano@unina.it (V.R.); immacolata.belviso@unina.it (I.B.); alessandro.venuta@unina.it (A.V.); montagna@unina.it (S.M.); 2 Department of Molecular Medicine and Medical Biotechnology, University of Naples Federico II, 80131 Naples, Italy; mariarosaria.ruocco2@unina.it (M.R.R.); federica.aliotta@unina.it (F.A.); 3 Department of Clinical Medicine and Surgery, University of Naples Federico II, 80131 Naples, Italy; stefania.masone@unina.it; 4 Department of Experimental and Clinical Medicine, University “Magna Graecia” of Catanzaro, 88100 Catanzaro, Italy; fiume@unicz.it; 5 Department of Structures for Engineering and Architecture, University of Napoli Federico II, 80125 Naples, Italy

**Keywords:** melanoma, tumor microenvironment, fibroblasts, melanoma-associated fibroblasts

## Abstract

Cutaneous melanoma (CM) tissue represents a network constituted by cancer cells and tumor microenvironment (TME). A key feature of CM is the high structural and cellular plasticity of TME, allowing its evolution with disease and adaptation to cancer cell and environmental alterations. In particular, during melanoma development and progression each component of TME by interacting with each other and with cancer cells is subjected to dramatic structural and cellular modifications. These alterations affect extracellular matrix (ECM) remodelling, phenotypic profile of stromal cells, cancer growth and therapeutic response. The stromal fibroblast populations of the TME include normal fibroblasts and melanoma-associated fibroblasts (MAFs) that are highly abundant and flexible cell types interacting with melanoma and stromal cells and differently influencing CM outcomes. The shift from the normal microenvironment to TME and from normal fibroblasts to MAFs deeply sustains CM growth. Hence, in this article we review the features of the normal microenvironment and TME and describe the phenotypic plasticity of normal dermal fibroblasts and MAFs, highlighting their roles in normal skin homeostasis and TME regulation. Moreover, we discuss the influence of MAFs and their secretory profiles on TME remodelling, melanoma progression, targeted therapy resistance and immunosurveillance, highlighting the cellular interactions, the signalling pathways and molecules involved in these processes.

## 1. Introduction

Cutaneous melanoma (CM) is the most aggressive skin cancer and accounts for 80% of skin cancer deaths and about 1–2% of all cancer deaths [1,2]. The development and progression of CM are characterized by three distinct steps: Radial Growth Phase (RPG) where cancer cells localize only to the epidermic layer, RGP-confined microinvasive, typical of CM containing some malignant cells in the superficial papillary dermis and Vertical Growth Phase (VGP) representing the tumorigenic and/or mitogenic phase of melanoma [1]. During the VGP step, CM can metastasize to lymph nodes, brain, lung, bone, and liver even if the size of the primary tumor is still small [3]. The high capacity of CM to disseminate, develop drug resistance, and hamper immunosurveillance depends on the heterogeneity of the cancer tissue composed of malignant cells and a tumor microenvironment (TME) [1,4,5]. In particular, TME includes extracellular matrix (ECM) molecules, growth factors, nutrients, blood and lymphatic tumor vessels and stromal cells represented by endothelial cells, pericytes, immune cells, fibroblast cell populations, activated adipocytes, and mesenchymal stem cells (MSCs) [1]. The cellular components of the TME are characterized by impressive phenotypic plasticity sustained by crosstalk with each other and with melanoma cells and involved in the regulation of cancer growth, targeted therapy resistance and immunosurveillance [1,3]. In this scenario, it is important to note that the transition from the normal dermal microenvironment, regulating skin homeostasis, to TME, is a crucial process affecting CM development and it is influenced mostly by stromal fibroblast populations [1,2,5,6,7]. The heterogeneous and plastic fibroblast populations can shift from an inactivated phenotype of normal quiescent fibroblasts either to an activated phenotype of normal myofibroblasts or constitutively activated phenotype of melanoma-associated fibroblasts (MAFs) and thus influence differently CM development and outcome [2]. In particular, the interaction of normal fibroblasts with melanoma cells leads to MAF differentiation, remodelling of the normal dermal microenvironment and its transformation to TME. MAFs represent the most abundant stromal cells of the TME and contribute dramatically to structural alterations of the microenvironment and molecular and cellular changes associated with CM outcome [2]. In particular, MAF secretory profiles, regulated by interactions of MAFs with cancer cells, influence significantly CM outcome [1,8]. Therefore, in this article we describe the biological role of fibroblast populations in the regulation of the normal skin microenvironment and TME and review the differences between normal fibroblasts and MAFs, highlighting their role in melanoma development. In particular, we discuss the influence of MAF different soluble and non-soluble factors on melanoma growth, ECM remodelling, targeted therapy resistance and immunosurveillance regulation. The deep understanding of signalling pathways regulating the flexible phenotype and secretory profiles of fibroblast populations, their interaction with cancer and stromal cells could be useful to develop therapeutic strategies targeting the TME and its pro-tumorigenic capability.

## 2. Normal Skin Structure and Melanoma Development: From Normal Dermal Microenvironment to Melanoma Microenvironment

In physiological conditions, structure and homeostasis of skin are highly controlled and maintained by dynamic interactions between normal melanocytes and the surrounding normal microenvironment, including keratinocytes, fibroblasts, endothelial, and immune cells and ECM [8]. These intercellular communications can take place through paracrine interactions, and/or cell–cell contact via cell adhesion molecules [9]. Normal melanocyte resides in the basal layer of the epidermis, where it makes contacts with thirty-six keratinocytes to form the “epidermal melanin unit” [10]. The “epidermal melanin unit” is a structural and functional unit regulating pigmentation and homeostasis of the epidermis [11]. Within the “epidermal melanin units”, keratinocytes tightly control melanocyte proliferation, and activity through paracrine interactions, and cell–cell contacts, in order to maintain a constant keratinocyte/melanocyte ratio [12]. Cell–cell contacts via adhesion molecules are crucial for the maintenance of the physiological position of melanocytes in the basal layer of the epidermis [13,14,15]. In fact, downregulation of cell adhesion molecules, such as E-cadherin, P-cadherin, desmoglein, and connexins, occurs during the malignant transformation of melanocytes and allows cancer cells to evade keratinocyte-mediated control [15,16], and acquire a higher invasive and metastatic capability [14,17,18]. Within normal skin, unlike keratinocytes, stromal fibroblasts are located in the dermis and do not physically interact with melanocytes. However, it is known that fibroblasts, as well as keratinocytes, exert a tight control of melanocyte growth and function by releasing soluble factors, such as stem cell factor (SCF) and neuregulin 1 (NRG1) [19,20,21]. During malignant transformation, the structural and functional organization of the “epidermal melanin unit” is gradually lost. Cell–cell adhesion and communication between melanocytes and normal cells in their immediate microenvironment are altered and the normal skin microenvironment is gradually rearranged and replaced by the TME [13]. Melanoma microenvironment appears as a complex, dynamic, and ever-changing mass, consisting of non-cancerous cells, such as stromal and immune cells, ECM proteins, growth factors, nutrients, blood and lymphatic tumor vessels. Each component of the melanoma microenvironment affects each other and deeply influences tumor growth, progression and therapy response, either favouring or preventing melanomagenesis [1]. Malignant melanocytes lose their preferential interactions with epidermal keratinocytes [22], proliferate along the dermo-epidermal junction and change their proper location by moving vertically up into the epidermis and then into the dermis [13]. Loss of E-cadherin appears to be one of the critical steps in melanoma progression [23]. In particular, the shift from E-cadherin to N-cadherin expression in malignant melanocytes uncouples melanocytes from keratinocytes and allows malignant cells to escape from keratinocyte control [24], and interact with N-cadherin-expressing cells, such as fibroblasts and vascular endothelial cells, in the dermis [22,23]. N-cadherin-mediated cell–cell adhesion is known to support cell survival by activating AKT signalling pathway, leading to increase in β-catenin levels and inactivation of the pro-apoptotic factor BAD [25]. Hence, the formation of new cell–cell contacts between malignant melanocytes and fibroblasts provides growth and metastatic advantages, by facilitating melanoma cell survival and transition from RGP to VGP. At this stage melanoma cells become competent to invade and metastasize to distant organs [24].

Of note, just as alterations in cell–cell interactions affect melanoma behaviour, alterations in cell–ECM communications can also act as driving forces of melanoma progression. The ECM is one of the major components of the TME. It provides necessary biochemical and biophysical cues to promote tumor cell proliferation, migration, and invasion [26,27,28,29]. Increased production of ECM proteins, such as *g*amma 2 chain of laminin 5 (laminin 5 γ2) [30], hyaluronan [31], collagen I [32,33], biglycan [34], tenascin-C and fibronectin [35] are associated with melanoma progression. Integrins represent the main transmembrane receptors used by cells to bind and respond to ECM proteins [36]. Of note, altered expression of integrins allows melanoma cells to escape from the primary tumor site, alter their cytoskeletal structure, migrate through the surrounding environment, and then eventually reach distant organs [9]. In particular, melanoma progression is associated with enhanced expression of β3 [37,38,39], and α2β1 integrins [40], as well as decreased expression of α4β1 [41], α6β1 [42], and α7β1 integrins [43,44]. The α_v_β_3_ integrin overexpression in melanoma cells is associated with induction of bcl2 to prevent apoptosis, and matrix metalloproteinase (MMP)-2 to break down the collagen of the basement membrane. These events enable melanoma progression and transition from RGP to VGP [38,45,46]. Furthermore, the upregulation of the α3β1 integrin on melanoma cell surface promotes cancer cell binding to laminin 5 [47], which is one of the major components of dermal ECM localized in the epidermal basement membranes [48]. Interestingly, an in vitro study reported that the binding of laminin-5 to melanoma cells enhances the production of MMP-9 (type IV collagenase), which is thought to be fundamental for invasive cells to degrade the basement membrane, invade, and thus modify the surrounding tumor stroma [47]. The tumor stroma is a crucial component of the TME and comprises all non-transformed tissue components associated with the tumor, such as several fibroblast populations, including fibroblasts, fibroblast aggregates, myofibroblasts, and cancer-associated fibroblasts (CAFs) [22,49,50,51,52,53,54]. The tumor stroma, whose composition varies significantly between different tumor types and during cancer evolution [55], deeply influences many hallmarks of cancer [56]. During RGP, CM is usually characterized by less evident fibroplasia and lymphocyte infiltration, while dysplastic nevi are associated with lamellar fibroplasia. During melanoma transition towards the VGP, concentric fibroplasia surrounds tumor cell nests [57,58]. Therefore, melanoma stroma can show signs of fibroplasia with desmoplastic characteristics for the extensive accumulation of fibroblasts, fibrocytes, and fibrillar ECM components [59]. Each component of the tumor stroma coevolves with cancer disease to accommodate the needs of the ever-changing neighboring tumor cells [60]. Therefore, during tumor development, not only melanoma cells but also stromal cells, including fibroblasts, undergo several molecular and functional changes, which could render them more capable of supporting tumor growth and progression [60].

The melanoma microenvironment is also characterized by a complex tumor vascular network, comprising regions of hypervascularisation and hypovascularisation [61]. In the TME, the increased number of tumor endothelial cells leads to the formation of tortuous, more permeable, dilated and saccular blood vessels [62]. The enhanced permeability of tumor vessels can lower fluid pressure gradient, and thus impair the delivery of nutrients, and oxygen in the tumor mass [62]. Impairment of oxygen delivery as well as the high oxygen consumption by cancer and endothelial cells generate hypoxic areas, which in turn promote the switch from oxidative phosphorylation (OXPHOS) to glycolytic pathways leading to acidosis [63]. Extracellular acidification of the TME and the peritumoral area is another important feature of melanoma [64]. Melanoma cells exposed to an acidic pH in vitro show enhanced secretion of proteinases and proangiogenic factors, increased invasive and angiogenic potential, and increased capability to develop metastases in vivo [65]. The extracellular acidosis of melanoma mainly depends on alterations of cancer cell metabolism [3]. One of the main metabolic alterations in melanoma cells, common to many cancer cell types, is the upregulation of aerobic glycolysis [3]. Indeed, melanoma cells use more glucose and generate more lactate than normal melanocytes [66]. The high amounts of lactate produced by melanoma cells are released into the extracellular environment and contribute to the acidification of the TME, and to the generation of an immunosuppressive niche, where T cells lose their tumor-killing ability [3,67,68]. Furthermore, the high rates of glucose uptake and increased glycolysis in melanoma cells create a glucose-deficient microenvironment, where glycolytic cancer cells and tumor-infiltrating lymphocytes (TILs) compete for glucose uptake. Hence, TILs exposed to low extracellular glucose, reduce the rate of glycolysis and become anergic [69]. The metabolic competition between melanoma cells and TILs results in a tolerogenic and immunosuppressive melanoma microenvironment [3].

## 3. Dermal Fibroblasts in Normal Skin: Features and Functions before Melanoma Occurrence

Human fibroblasts are the predominant stromal cell type of the connective tissue [70], and the main effector of many physiological and pathological processes, such as ECM turnover and homeostasis [71], epidermal regeneration [72], wound healing and tumor [73,74]. The dermis of normal skin consists of two different fibroblast subpopulations with distinct cell morphology, gene expression patterns, and capability [75,76]. Reticular and papillary fibroblasts, populating the lower and upper layers of the dermis, respectively, act together, differently and sequentially in order to guarantee the maintenance of skin physiology, and the regulation of cutaneous wound repair [77,78]. In healthy skin tissues, in the absence of injury, normal dermal fibroblasts persist in a quiescent state [79]. Conversely, in response to cutaneous injury, occurring for instance during wound healing, normal dermal fibroblasts experience dramatic molecular and functional changes. Mechanical stress and specific factors allow quiescent dermal fibroblasts to transiently differentiate into myofibroblasts [79,80,81], which are characterized by an activated phenotype, associated with de novo expression of alpha-smooth muscle actin (α-SMA) [82], higher cellular contractility, and increased ability to promote ECM synthesis and wound closure [83,84]. In particular, reticular dermal fibroblasts migrate into the wounded site early, generating a collagen-rich dermis resembling a scar [85]. Their main function is the regulation of cytoskeletal organization and cell motility [76]. Conversely, papillary dermal fibroblasts play a fundamental role in the final stage of wound healing [86]. They are mainly involved in the formation of the basement membrane, keratinocyte growth [87] and immune response [76]. The normal wound healing process ends with wound closure and activated dermal fibroblast disappearance from the healed wound. In fact, it has been widely demonstrated that normal myofibroblasts disappear after wound resolution by either returning back to a quiescent state or undergoing cell death via apoptosis [88]. Conversely, in cancer, which is described as a “wound that does not heal” [89], fibroblasts can acquire a constitutive activated phenotype, resistance to apoptotic stimuli, and thus differentiate into the so-called CAFs with tumor-promoting capability [90]. Interestingly Hogervorst et al. demonstrated that reticular fibroblasts are more predisposed to differentiate into CAFs and promote tumor invasion and epithelial to mesenchymal transition (EMT) than papillary fibroblasts [91]. Therefore, taken together, all these data could indicate that distinct functions of reticular and papillary fibroblasts observed during the wound healing process, may reflect their capability to generate different TMEs that differently affect cancer progression.

## 4. Fibroblast Populations of Melanoma Microenvironment

### 4.1. Normal Dermal Fibroblasts at the Onset of Melanoma

**Figure 1:**
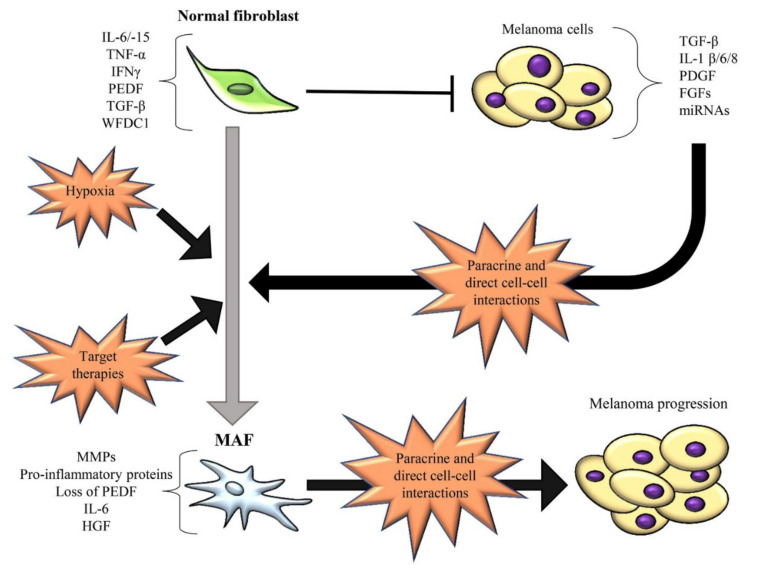
Schematic representation of the bidirectional interactions between normal fibroblasts or melanoma-associated fibroblasts (MAFs) and melanoma cells. The main soluble mediators involving in this crosstalk are reported. Normal fibroblasts can inhibit the growth of melanoma cells. IL-6/-15, TGF-β, PEDF, TNF-α, IFNγ and WFDC1 are produced by normal fibroblasts and associated with melanoma inhibition. Concurrently, normal fibroblasts are forced by melanoma cells to acquire a constitutively activated state and differentiate into the tumor-promoting MAFs. MAF differentiation occurs in the tumor mass through melanoma cell-derived soluble factors (such as TGF-β, IL-1β, PDGF, FGF, miR-155/-210/-211) or direct cell–cell contacts. In turn, MAFs secrete soluble mediators (such as pro-inflammatory proteins, MMPs, IL-6, HGF, etc.) leading to melanoma growth and progression. Of note, IL-6 and TGF-β act as tumor suppressors at early stage of melanoma and tumor promoters at late stage. Other conditions promoting MAF differentiation are represented by hypoxia and target therapies (such as MAPKi). Black T bar represents inhibition, while black arrows depict induction. The transition from normal fibroblasts to the tumor-promoting MAFs is indicated by the grey arrow.

It is noteworthy that before CAF differentiation, fibroblasts can hamper melanoma initiation and progression (Figure 1).

Indeed, normal dermal fibroblasts can act as physical and signalling barriers to impede melanoma initiation before melanoma cells stimulate them to become CAFs [92]. Cornil et al. demonstrated that normal dermal fibroblasts exert an in vitro inhibitory effect on the growth of several melanoma cell lines derived from early-stage RGP or VGP [93]. Another in vitro study further demonstrated that A375 and A2058 melanoma cell viability and migration are significantly reduced by the conditioned media derived from normal fibroblasts [54]. However, the molecular mechanisms and factors used by normal dermal fibroblasts to interact with melanoma cells and block tumor progression are still poorly understood. By using a melanoma mouse model, Zhou et al. demonstrated that normal dermal fibroblasts inhibit the EMT and induce a G1/S cell cycle arrest in melanoma cells through the MAPK/ERK and Rb signalling pathways [92]. One study identified interleukin (IL)-6 as a fibroblast-derived factor involved in the growth inhibition of melanoma cells derived from RGP or early VGP primary lesions. Conversely, melanoma cells obtained from advanced VGP and distant metastatic lesions were shown to be completely resistant to IL-6-mediated growth inhibition [94]. Therefore, it is possible to assume that the anti-tumor role exerted by dermal fibroblasts could be associated with their ability to produce anti-tumor proteins (Figure 1), such as transforming growth factor-beta (TGF-β), which can act as a tumor suppressor at the early stages of carcinogenesis [22,95], whey acidic protein four-disulfide core domain 1 (WFDC1) [96] and several cytokines, such as interferon-gamma (IFNγ), tumor necrosis factor-alpha (TNF-α) and IL-15, which are involved in immune cell mobilization [97,98]. Furthermore, normal fibroblasts can counteract the degradation of the basement membrane and therefore prevent melanoma cell invasion into the dermis thanks to their ability to regulate and restrain ECM changes in healthy skin tissue. In particular, they exert a strictly control of MMP-1,-2,-9, and -13, and membrane-type matrix metalloprotineases (MT-MMPs) expression [22,99]. Anyway, it is important to note that during melanoma development, the reciprocal and continuous interactions between melanoma cells and fibroblasts determine profound molecular and functional changes in both the tumor and the stromal compartments. Consequently, despite their initial anti-tumor role, normal dermal fibroblasts are continuously forced by melanoma cells to acquire a constitutive myofibroblastic phenotype and thus differentiate into MAFs with pro-tumor capability [1,100] (Figure 1).

### 4.2. Origins and Features of Melanoma-Associated Fibroblasts

MAFs represent one of the most important non-cancerous stromal cell types within melanoma microenvironment with pro-tumor functions. MAFs, as well as other CAFs, share many similarities with myofibroblasts found during wound healing and inflammatory process [1,3,53]. Indeed, MAFs express high levels of α-SMA, which is one of the most significant markers of fibroblast activation and CAF differentiation [73,101,102,103]. In addition to α-SMA, fibroblast activating protein (FAP), fibroblast specific protein 1 (FSP-1), osteonectin, desmin, platelet-derived growth factor receptor (PDGFR), podoplanin (PDPN), periostin (POSTN), neuron-glial antigen-2 (NG2), CD90/THY1 and the mesenchymal protein vimentin are highly expressed by CAFs and thus used as CAF markers [73,104,105]. However, all these markers are not exclusively and synchronously expressed by CAFs. Different CAF profiles define distinct CAF subsets with different functions. Wong et al. stratified MAF populations in terms of their expression of Thy1, smooth muscle actin (SMA), and FAP markers and demonstrated in pre-treatment melanoma specimens that MAF profiles are associated with melanoma immunotherapy outcome. In particular, in anti-PD-1 treated melanoma patients, both high Thy1 and high FAP cell counts are associated with increased overall survival, whereas SMA cell count shows negative associations with patient outcome and survival. Hence, the differences in survival associations for SMA, Thy1 and FAP markers reflect the molecular and functional complexity of MAF subsets. Interestingly, FAP is inversely associated with prognosis in a historical cohort of melanoma patients that did not receive immunotherapy. However, further studies are needed to better clarify the role of FAP in immunotherapy. Taken together all these results suggest that multiplex MAF profiling can support immuno-oncology by facilitating the identification of melanoma patients who will benefit or not from anti-PD-1 therapy [106].

MAFs, as well as other CAFs, can originate from distinct cell populations, including epithelial cells via EMT, endothelial cells via endothelial-to-mesenchymal transition (EndMT), MSCs, fibrocytes, pericytes, smooth muscle cells, and adipocytes through transdifferentiation [73,107]. Interestingly, MAFs can also arise from melanoma cells differentiating towards a CAF/myofibroblast-like phenotype by the paradoxical effect of MAPK-targeted therapies [108]. Girard et al. demonstrated that MITF^low^/AXL^high^ melanoma cells resistant to BRAF inhibitors (BRAFi) exhibit a CAF-like phenotype due to their acquired ability to deposit a fibrillar ECM network, composed of collagen fibers, collagen cross-linking enzymes, fibronectin, tenascin C, and thrombospondin 1 [109]. Another study showed that after short-term BRAF inhibition, PTEN-null melanoma cells acquire a CAF-like phenotype, especially regarding their ability to generate a fibronectin-derived protective niche, that allows therapeutic escape [108,110]. However, pre-existing resident dermal fibroblasts undoubtedly represent the primary source of CAFs in melanoma [22]. Upon continuous paracrine and/or cell–cell interactions with melanoma cells, normal dermal fibroblasts gradually acquire a cancer-promoting phenotype [1] (Figure 1), characterized by a constitutive activated state and increased ability to promote tumor growth, invasion, aggressiveness [111] and drug tolerance [112]. In vitro co-culture experiments showed that the bidirectional interplay between fibroblasts and melanoma cells provokes dramatic alterations in fibroblast gene expression pattern and a more modest effect in melanoma cell gene expression profile. In particular, co-cultured fibroblasts were shown to upregulate genes associated with matrix degradation, such as MMP-1 and MMP-3, cell proliferation, and proinflammatory pathways, including IL-1β, IL-8, GROβ, GROα, and CCL2 [100]. Several studies have pointed to identify specific factors and/or molecular mechanisms involved in MAF differentiation (Figure 1). Whipple et al. reported that BRAF^V600E^ melanoma cells secrete high levels of cytokines, such as IL-1β, IL-6, and IL-8, which favour stromal fibroblast activation (Figure 1). Among these cytokines, IL-1β was identified as a central melanoma cell-secreted factor involved in fibroblast activation and CAF differentiation [113]. IL-1 and bFGF produced by melanoma cells represent possible mediators responsible for the expression of MMP-1, whose levels are higher in MAFs than normal fibroblasts [114]. Other important factors involved in fibroblast activation and MAF formation are represented by TGF-β, PDGF, FGF-2 and FGF-19 [115] (Figure 1), which are highly expressed by melanoma cells [116,117,118]. In particular, it is noteworthy that TGF-β acts as a negative growth factor for epithelial cells and normal melanocytes, but not for melanoma cells, which develop resistance to TGF-β-induced inhibition [8,119]. TGF-β1, frequently found increased in sera of patients with malignant melanoma [102,120], is used by melanoma cells to modulate their surrounding stroma and stimulate the conversion of fibroblasts into α-SMA-expressing myofibroblasts and thus into CAFs (Figure 1) [101,121]. Ngwani et al. demonstrated that TGF-β released by melanoma cells sensitizes fibroblasts to platelet-derived growth factor-BB (PDGF-BB) by upregulating its receptor PDGFR-β on fibroblast surface and increasing PDGF-BB expression by fibroblasts. Consequently, stromal and tumor cell-derived PDGF-BB binds to fibroblast-expressing PDGFR and decreases pigment epithelium-derived factor (PEDF) expression in fibroblasts via JNK1/2 and p38 signalling pathway. Loss of PEDF in fibroblasts drives CAF conversion and enhances the expression of genes involved in tumor progression and metastasis, such as IL-8, SERPINB2, and hylauronan synthase. Of note, PEDF, highly expressed by normal fibroblasts, impedes CAF conversion. Consequently, Ngwani’s study suggests that the pharmacological inhibition of PDGF-BB and TGF-β, and the use of PEDF small peptide mimetics could be novel and promising therapeutic approaches to restore the in vivo expression of PEDF in fibroblasts and the tumor milieu and restrain or reverse fibroblast activation thus impeding CAF transformation [122].

Nodal, a member of the TGF superfamily that correlates with an increased level of α-SMA in melanoma lesions, is another factor driving fibroblast activation and CAF formation [123]. Yin et al. demonstrated that the inhibition of TGF-β signalling with TGF-β receptor-I/ALK5 inhibitor or TGF-β–neutralizing antibodies disallows the activation of fibroblasts and inhibits their ability to promote melanoma growth and invasion [124]. Therefore, this study suggests that the inhibition of TGF-β signalling pathway may support the disruption of the crosstalk between MAFs and melanoma cells, and consequently block melanoma progression. Furthermore, galectin-1, which is involved in the activation of dermal fibroblasts into myofibroblasts [125], and is highly expressed in melanomas and particularly in advanced lesions [126], could represent another factor involved in MAF activation and differentiation.

It has been increasingly recognized that microvesicles (MVs) secreted by cancer cells are potent mediators of a wide range of biological and cellular functions, including tumor-stroma communication and transformation of normal fibroblasts into MAFs. In particular, Zhao et al. demonstrated that hypoxia, which is one of the main features of melanoma microenvironment [3], increases tumor MV secretion and the expression of the integrin very late antigen 4 (VLA-4) on melanoma cells. Furthermore, they reported that MVs from hypoxia-stimulated melanoma cells enhance fibroblast expression of IL-6, FAP and EGF, which are CAF markers. Of note, MV-educated fibroblasts are also characterized by enhanced expression of vascular cell adhesion molecule-1 (VCAM-1) [127], which is the major ligand of VLA-4 [128]. Therefore, the bidirectional upregulation of VLA-4 on melanoma cells and VCAM-1 on fibroblasts via tumor MVs and hypoxia facilitates the adherence of cancer cells to stromal cells and consequently triggers melanoma progression [127]. The synergy between hypoxia and stromal fibroblasts in melanoma progression has been further highlighted by the study of Chiarugi‘s group. They demonstrated that hypoxia is one of the most active factors responsible for the activation of dermal fibroblasts into myofibroblasts. Interestingly, they showed that hypoxic myofibroblasts exploit hypoxic oxidative stress to enhance melanoma invasion and chemotaxis [129].

Interestingly, Dror et al. demonstrated that CAF formation from local dermal fibroblasts can occur before melanoma spread into the dermis. This differentiation is stimulated by melanosomes, which are pigment vesicles transferred from melanoma cells located in the epidermis to normal fibroblasts located in the dermis. Consequently, this paracrine and distant communication allows the formation of a dermal tumor niche, where fibroblasts are reprogrammed into CAFs. In particular, the melanosomal miR-211 was identified as an important molecule involved in fibroblast differentiation into CAFs (Figure 1). Melanoma melanosomal miR-211 affects the expression of insulin-like growth factor 2 receptor (IGF2R), upregulate MAPK signalling pathway, which results in increased fibroblast proliferation, migration, and over-production of proinflammatory proteins, including IL-1β, IL-6, IL-8, CXCL1, CXCL2, and cyclooxygenase 2 (COX-2) [53]. Other miRNAs, that are delivered by human melanoma exosomes and involved in MAF differentiation, are represented by miR-155 and miR-210 (Figure 1). These miRNAs promote the metabolic reprogramming of normal dermal fibroblasts leading to the acquisition of a glycolytic phenotype and thus MAF generation. In particular, miR-155 upregulates glucose metabolism by increasing glycolysis while miR-210 decreases OXPHOS in fibroblasts [130]. MiR-155 is also involved in the proangiogenic switch of CAFs via suppression of SOCS1 expression, activation of the JAK2/STAT3 signalling pathway and overexpression of MMP-9, vascular endothelial growth factor (VEGF)-A, and FGF-2 [131].

It is noteworthy that although the use of MAPK inhibitors (MAPKi) in melanoma treatment represents an effective therapeutic strategy [3,112], complete remissions could be counteracted by the development of acquired resistance and MAPKi capacity to promote stromal remodelling and CAF activation [108,112]. Fedorenko et al. demonstrated that vemurafenib (MAPKi) can activate local fibroblasts through paradoxical induction of the MAPK signalling pathway, forcing them to differentiate into CAFs and enhance hepatocyte growth factor (HGF) expression. This evidence suggests that off-target effects of kinase inhibitors can remodel the host environment, which in turn can favour therapy resistance and thus drive melanoma progression. Therefore, MAF activity in the melanoma microenvironment should be considered to develop the most effective therapeutic strategy for melanoma treatment [132].

## 5. MAFs as a Key Driver of Melanoma Growth and Progression

### 5.1. MAF-Secreted Factors Involved in Melanoma Cell Proliferation and Migration

**Figure 2:**
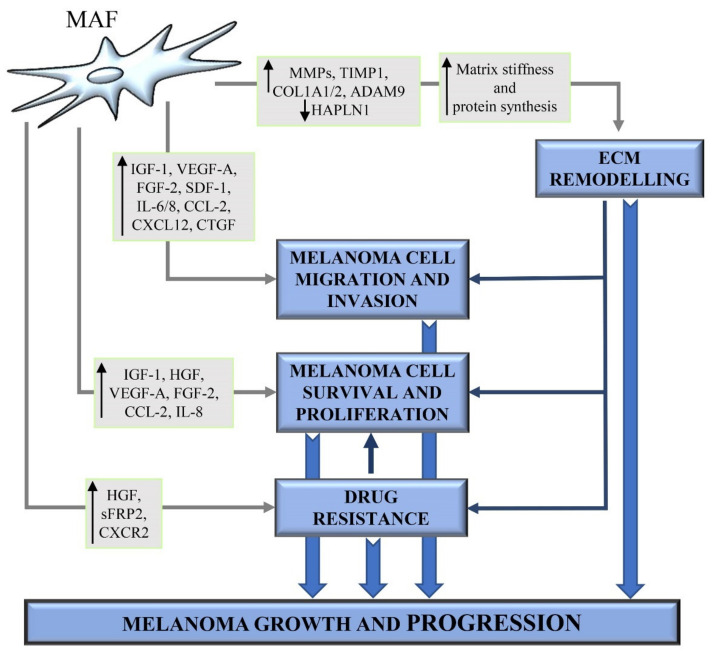
Involvement of MAFs in melanoma growth and progression. Schematic representation of the principal MAF-derived molecules, including growth factors, cytokines, chemokines and proteases leading to extracellular matrix (ECM) remodelling, pharmacological resistance, and increased melanoma cell survival, proliferation, and migration. MAF-derived ECM is a functional and structural support network, enabling melanoma cells to proliferate, survive also during pharmacological treatment, migrate, and escape from the primary tumor site to colonize distant sites. ECM proteins produced by MAFs induce pro-survival, pro-proliferative, and pro-migratory signalling pathways in melanoma cells.

The secretory profile of MAFs significantly affects melanoma growth and progression. MAFs synthesize and secrete large amounts of growth factors and cytokines in order to promote melanoma cell proliferation, migration and invasion (Figure 2) [129].

In particular, upon activation by melanoma cells, fibroblasts were shown to produce growth factors like insulin-like growth factor-1 (IGF-1) and HGF (Figure 2) [8]. IGF-1 increases the survival, growth, and migration of melanoma cells from early tumor lesions by activating MAPK and AKT signalling pathways. Melanoma cells from late primary or metastatic lesions show constitutive activation of the MAPK signalling pathways and a higher level of stabilized β-catenin, which is an important downstream molecule in the AKT signalling pathway. Therefore, at the late stage of melanoma development, melanoma cells are unresponsive to the growth stimulation by IGF-1 [133]. Satyamoorthy et al. further demonstrated that fibroblast-derived IGF-1 promotes IL-8 expression in melanoma cells, especially from early melanoma lesions, via activation of MAPK/JNK/c-Jun/AP-1 signalling pathway [23]. Blocking IL-8 signalling in melanoma cells was shown to abrogate IGF-1-induced melanoma cell migration [23]. HGF is a multifunctional cytokine highly produced by mesenchymal cells [8], including CAFs [22], melanoma cells, but not by normal melanocytes [8]. Several studies showed that HGF promotes melanoma progression by favouring the switch from E- to N-cadherin and downregulating desmoglein 1 expression [16,134]. Koefinger et al. demonstrated that E-cadherin to N-cadherin switch mediated by HGF is associated with stage-specific changes in the expression levels of the EMT regulators Snail, Slug and Twist [134]. Furthermore, it has been reported that CAF-derived HGF can promote melanoma progression by enhancing melanoma cell proliferation via tyrosyl-phosphorylation of MET, MAPK and ERK2 (Figure 2) [22,135]. CAFs are also stimulated by melanoma cells to increase the expression and secretion of large amounts of VEGF-A and FGF-2 (Figure 2) [131], whose levels in melanoma sera patients closely correlate with poor clinical outcomes [136]. VEGF-A and FGF-2 are known to act on the endothelial compartment, by promoting proliferation, survival, migration and tube formation of endothelial cells [131]. Furthermore, they also act on the tumor compartment by enhancing melanoma cell invasion, angiogenesis and brain metastasis [137].

Hypoxia, one of the main hallmarks of the melanoma microenvironment [3], enables dermal fibroblasts to enhance the secretion of VEGF-A, stromal-derived factor-1 (SDF-1) and IL-6, which cooperate to promote melanoma cell chemotaxis and invasion (Figure 2). Treatment with blocking antibodies targeting CXCR4, the receptor for SDF-1, VEGF-A or IL-6, has been shown to reduce melanoma cell invasiveness induced by the conditioned media from hypoxic activated fibroblasts [129]. Li et al. demonstrated that fibroblasts cocultured with invasive melanoma cell lines significantly upregulate the expression of genes encoding CCL2 and IL-8 (also known as CXCL8) (Figure 2) compared to fibroblasts cocultured with non-invasive melanoma cells [118]. CCL2 and IL-8 are two potent stimulators of angiogenesis, leading to enhanced tumor vascularization and improved oxygen and nutrient supply to cancer cells [138]. CCL2 and IL-8 have been also shown to induce tumor cell proliferation and EMT, respectively. All these events result in tumor progression and metastatic spread [138]. Furthermore, Jobe et al. demonstrated that IL-6 and IL-8, which are upregulated in sera of melanoma patients and correlate with their overall survival, are promising molecular targets to inhibit melanoma cell invasion successfully [139,140]. They demonstrated that CAF-derived conditioned medium promotes melanoma cell migration and invasion and that the simultaneous blocking of IL-6 and IL-8 with neutralising antibodies is sufficient to fully suppress CAF-induced human melanoma cell invasiveness [139,140].

CXCL12 is another pro-angiogenic cytokine highly secreted by MAFs [22,141]. In addition to recruiting endothelial cells into the tumor stroma, CXCL12 interacts with its receptor CXCR4 on the melanoma cell surface [142], and enhances melanoma cell adhesion to endothelial cells via an integrin β1-based mechanism, both in vitro and in vivo [143], and promotes melanoma cell migration from the blood to distant organs, especially to lungs [142].

The connective tissue growth factor (CTGF or CCN2) is a cysteine-rich matricellular protein [144,145] highly secreted into the ECM by both malignant melanoma cells and activated fibroblasts within the tumor mass (Figure 2) [145,146]. Although CTGF is not considered to be a structural component of the ECM, it significantly affects cellular behaviour by altering the TME and leading to cancer growth and metastasis. In particular, Hutchenreuther et al. demonstrated that MAF-derived CTGF contributes to melanoma invasion and metastasis (Figure 2), without affecting melanoma cell proliferation and cancer growth. Loss of fibroblast-derived CTGF reduces melanoma cell capability to invade through collagen in vitro and form metastases to the lungs in vivo. Therefore, MAF-derived CTGF blockade may represent a promising therapeutic strategy to mitigate melanoma progression [145].

### 5.2. MAF-Mediated Remodelling of the ECM

The ECM plays a crucial role in normal physiological conditions [147]. Changes in mechanical and biochemical properties of the ECM, aberrant and excessive ECM deposition and degradation characterize many pathological conditions, including melanoma [33]. CAFs represent the major player involved in the production and remodelling of the ECM in the tumor mass (Figure 2) [148]. Matrices derived from MAFs and normal dermal fibroblasts differ for biochemical and mechanical properties. MAFs generate ECM enriched in fibrillar collagens, with a high level of fiber organization, enhanced stiffness with respect to the ECM generated by normal dermal fibroblasts [149]. Throughout melanoma development, melanoma cells are exposed to various types of ECM, with collagen progressively becoming the predominant ECM proteins [33]. The collagens, often crosslinked and linearized, increase the stiffness of the TME. This stiffening can regulate phenotypic states and contribute to the acquisition of a malignant phenotype [150,151]. The importance of matrix stiffness in melanoma progression has been confirmed by several studies. Fibroblasts incubated with melanoma cell-conditioned medium generate collagen- and fibronectin-denser and stiffer ECM, where melanoma cells have increased cell viability [33]. Furthermore, it has been reported that the expression of biglycan, an important and abundant component of melanoma microenvironment, stimulates the formation of a dense collagen matrix, characterized by increased stiffness. This matrix stiffness promotes melanoma cell migration and invasion via upregulation of integrin-β1 on melanoma cell surface. Biglycan-deficient fibroblasts generate a less rigid matrix, with decreased expression of integrin-β1 and, consequently, a reduced capability to promote melanoma cell invasion [34]. Interestingly, Hirata et al. demonstrated that fibronectin-rich matrices with 3–12 kPa stiffness are sufficient to provide BRAFi PLX4720 resistance to melanoma cells. Conversely, melanoma cells plated on low stiffness collagen I show high levels of cell death following treatment with the BRAFi PLX4720 [152]. Therefore, taken together, all these data indicate that an appropriate matrix composition and stiffness can provide survival advantage and drug resistance to melanoma cells.

Upon stimulation with melanoma cell-conditioned media in vitro, fibroblasts increase gene expressions of matrix proteins, such as COL1A1 and COL1A2, as well as matrix remodelling proteins, such as MMP-2 and TIMP1 (Figure 2) [33]. In addition to MMP-2, CAFs secrete other proteolytic enzymes, including MMP-1, MMP-13, and MMP-14, which sustain melanoma invasion by leading to ECM digestion and formation of “tracks”, that melanoma cells use to move through the tumor mass and eventually leave the primary site [22,114,153,154].

ADAM-9 is a proteolytic and adhesive protein that allows cell–cell contacts between fibroblasts and melanoma cells. ADAM-9 also induces cell signalling leading to increased secretion of the proteolytic enzymes MMP-1 and MMP-2 in fibroblasts and MMP-2 in melanoma cells. Furthermore, ADAM-9 ablation in fibroblasts has been shown to strongly inhibit both cell–cell adhesion and melanoma cell invasion in vitro. Therefore, ADAM-9-mediated proteolytic activities of melanoma cells and stromal fibroblasts also contribute to ECM remodelling to produce a TME suitable for melanoma cell migration and invasion (Figure 2) [155]. Among melanoma cell-secreted factors, TGF-β plays a fundamental role also in ECM production and remodelling by CAFs. Indeed, it has been reported that the paracrine effects of TGF-β on fibroblasts within the melanoma microenvironment enable MAFs to increase production and deposition of ECM proteins, which thus generate a supportive scaffold for melanoma cell survival and proliferation (Figure 2). Additionally, in vivo data demonstrated that MAFs surrounding TGF-β-producing melanoma cells increase collagen, fibronectin, tenascin, and α2 integrin production. Microarray studies further confirmed the in vivo results by highlighting an increase of tenascin and types VI, XV, and XVIII collagen expression in TGF-β1-transduced fibroblasts [121]. The CAF marker FAP, highly expressed by reactive stromal fibroblasts of both primary and metastatic melanomas, is a serine protease having both dipeptidyl peptidase and collagenolytic activities and responsible for the degradation of gelatin and type I collagen [156]. Therefore, FAP enzyme activity detected in extracts of melanocytic nevi and melanoma metastases but not in normal adult skin [156], can contribute to ECM remodelling and facilitate cancer cell growth and migration [157,158]. Furthermore, in melanoma, aged fibroblasts express low levels of hyaluronan and proteoglycan link protein 1 (HAPLN1), which is an ECM-modifying protein involved in the cross-linking of hyaluronan to the ECM. Loss of HAPLN1 triggers the breakdown in the cross-linking of the ECM, and destabilizes lymphatic vessel integrity, leading to increased permeability. The increase of lymphatic endothelial permeability, due to HAPLN1 loss, promotes migration and invasion of melanoma cells, and their escape from the lymphatic system to distant metastatic sites (Figure 2). These data could have profound implications for the treatment and surveillance of elderly patients with melanoma [159,160]. Furthermore, it is noteworthy that CAFs generate a therapeutic “safe-haven” for melanoma cells treated with MAPKi by remodelling the ECM [152]. However, specific mechanisms activated by MAF-derived matrix and leading to melanoma MAPKi resistance will be discussed later.

### 5.3. MAF-Immune Modulating Functions

**Figure 3:**
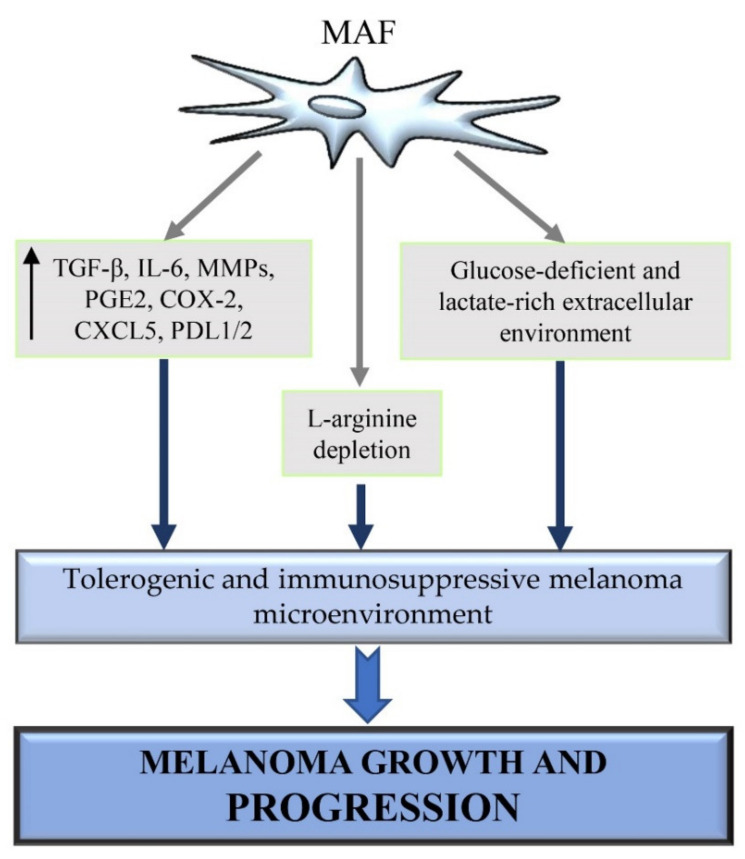
MAF immunomodulatory functions. MAFs generate an immunosuppressed melanoma microenvironment by multiple mechanisms. They increase the expression of various inflammatory and immunosuppressive factors, including TGF-β, IL-6, MMPs, PGE2, COX-2, CXCL5, and PDL1/2, which dramatically impair the anti-tumor activity of immune cells. Furthermore, MAFs alter the extracellular availability of lactate, glucose, and arginine, which are important immune-modulating metabolites involved in immune cell suppression or polarization toward a tumor-promoting phenotype. Consequently, the generation of an immunosuppressive, glucose- and arginine-poor, lactate-rich melanoma microenvironment allows melanoma cells to evade immune surveillance and thus survive and proliferate safely in the tumor mass.

The immune system plays a pivotal role in melanomagenesis because it may either contribute to the eradication of cancer or potentiate melanoma growth and tumor cell proliferation [161]. The dual role exerted by the immune system during the progression of many solid tumors, including melanoma, can be explained by the so-called cancer immune editing. Cancer immune editing is a dynamic process allowing immune cells to switch from an anti-tumor immune state to a pro-tumor immune state. In particular, immune editing is characterized by an initial elimination phase where immune cells destroy cancer cells by the innate immunity effectors [162,163]. Interestingly, spontaneous regression occurs more frequently in melanoma than in other tumors. Melanoma regression is caused by an efficient immune response against melanoma cells [164,165]. However, the elimination phase can be followed by the so-called equilibrium phase. It is the longest phase, lasting also for years, during which the tumor cells are constantly suppressed whereas resistant neoplastic variants are generated [162,163]. During the last phase of cancer immune editing, known as the immune escape phase, neoplastic variants become more resistant to the identification and/or elimination by the immune system, leading to tumor growth and progression [166]. The immune escape is associated with the functional exhaustion of the immune cells and with the induction of events altering the immune-mediated recognition of cancer cells [167]. CAFs have been shown to play a pivotal role in the regulation of both the innate and the adaptive tumor immune response by releasing pro-inflammatory and immunosuppressive factors in the TME [168,169]. For instance, immunosuppressive cytokines, including TGF-β, IL-6; and VEGF [166], are known to be overexpressed by MAFs [53,101]. Therefore, the release of these cytokines and other molecules by MAFs may favour MAF’s capability to mobilise immunosuppressive cells and thus generate an immunosuppressive environment, that is crucial for tumor growth and progression (Figure 3).

It is known that TGF-β can inhibit migration, maturation, and antigen presentation by dendritic cells, enhance the number of regulatory T cells (Tregs) within the TME and decrease the expression of granzymes, perforin, FAS ligand (FASL), and IFNγ in cytotoxic T cells [170,171,172]. Furthermore, it has been reported that TGF-β can also drive resistance to PD-1 inhibitors and contribute to the downregulation of the major histocompatibility complex (MHC) class I in melanoma cells [173]. IL-6, highly produced also by MAFs [53,127], can promote the expression of the immunomodulatory cytokine IL-10 in melanoma cells. Melanoma derived-IL-10 reduces immune response by suppressing the function of antigen-presenting cells (APCs), blocking the production of pro-inflammatory cytokines, and downregulating the expression of co-stimulatory molecules and MHC class II [174,175].

Ziani et al. demonstrated that MAFs protect melanoma cells against NK cell-mediated cytotoxicity by the secretion of high levels of active MMPs (Figure 3). The release of active MMPs by MAFs decreases the expression of the two NKG2D ligands, MICA/B, on melanoma cell surfaces and consequently reduces the NKG2D-dependent cytotoxic activity of NK cells against melanoma cells [176]. Furthermore, it has been demonstrated that stromal fibroblast-derived MMP-9, involved in the cleavage of PD-L1 from melanoma cell surface, leads to anti-PD-1 therapy resistance in melanoma. The silencing of MAF MMP-9 expression reverses MAF capability to inhibit anti–PD-1 responses and increases the ratio of CD8+ T cell/Treg in melanoma in vivo [177]. MAFs can profoundly alter the NK-dependent anti-tumor immune response also by releasing prostaglandin E2 (PGE2) (Figure 3). In particular, by producing PGE2 either constitutively or upon induction by NK cells, MAFs decrease the expression of two activating NK-receptors, NKp44 and NKp30, at the surface of NK cells, leading to an impairment of the NK cell-mediated killing of melanoma cells. Furthermore, transwell cocultures and the use of specific inhibitors showed that the direct interactions between MAFs and NK cells are important to inhibit DNAM-1 expression in NK cells [178]. DNAM-1 is an adhesion molecule expressed by NK cells and involved in the induction of NK cell cytotoxicity [179]. Immunohistochemistry analysis of melanoma specimens revealed that MAF/NK cell interaction could also occur in vivo within the tumor mass and lead to NK cell impairment via cell–cell interaction. In fact, NK cells were detected in close vicinity to MAFs surrounding melanoma metastatic lesions [178]. Therefore, NK cell killing capability is associated with the expression of NK cell receptors, such as NKp44, NKp30 and DNAM-1, able to recognize and bind to ligands expressed by melanoma cells [178]. Furthermore, it has been shown that mutation in BRAF induces the production of IL-1α and –β in melanoma cells, which in turn enhances MAF’s capability to suppress the proliferation and function of cytotoxic T cells. In particular, MAFs exposed to tumor-derived IL-1 α/β induce T cell suppression by rapidly upregulating the expression of PD-1 ligands (PD-L1 and PD-L2) and COX-2 [180], which are well-known molecules exerting powerful suppressive effects on T cells, in multiple cancer types (Figure 3) [181,182]. α-SMA^+^ CAFs from melanoma, colon and lung cancers, have been shown to express the immune checkpoint molecules PD-L1 and PD-L2, which strongly induce T cell exhaustion [180,183,184]. Furthermore, Li et al. demonstrated that α-SMA^+^ CAFs from melanoma and colorectal carcinoma, can also increase PD-L1 expression in tumor cells via CX-chemokine ligand 5 (CXCL5) secretion and activation of PI3K/AKT signalling pathway (Figure 3) [185]. Interestingly, the neutralization of PD-1 ligands and COX-2 were shown to partly reduces MAF-mediated T cell suppression, whereas the combination of IL-1 α/β and PD-1 ligands neutralization with COX-2 inhibition enhances T-cell cytokine production even further [180]. Furthermore, the in vivo localization of MAFs frequently lining the tumor vasculature and/or forming a physical barrier between TILs and cancer cells, could indicate that MAFs are located ideally to promote immune cell suppression in vivo [180]. Interestingly, analysis of CAFs from lung adenocarcinomas and melanomas revealed that this stromal cell type is also able to process and present antigens, leading to antigen-specific deletion of cytotoxic T cells and enhanced cancer cell survival. After antigen presentation, CAFs directly interact with activated CD8+ T cells, thus promoting T cell dysfunction and death via PD-L2 and FASL engagement [186]. Furthermore, Érsek et al. demonstrated that MAFs can hamper cytotoxic T cell-mediated killing by altering ERK1/2 and NF-κB signalling pathways, impeding the expression of the early T cell activation marker CD69 and granzyme B production, and enhancing the expression of two potent negative regulators of T cell activity, T cell immunoreceptor with Ig and ITIM domains (TIGIT) and B and T lymphocyte attenuator (BTLA) [187]. As previously discussed, the reduction of glucose availability in the melanoma microenvironment is associated with the generation of a glucose-deficient and immunosuppressive milieu, where different cells compete for glucose uptake [3]. In melanoma microenvironment, melanoma cells as well as MAFs show a highly glycolytic phenotype and increased glucose avidity [3,188]. Of note, also T cells highly depend on glycolysis for their high energetic demand during proliferation and cytokine production [68]. Therefore, in the tumor mass, the continuous crosstalk and competition for glucose uptake lead T cells to be deprived of glucose and increase several “anergy” signature gene expression. These extracellular and intracellular events drive loss of the tumor-killing ability by T cells [3]. In addition to the increase in glycolytic rate and glucose uptake, MAFs also show a reduction in oxygen consumption and a massive increase in lactate production and secretion (Figure 3) [188]. It is noteworthy that lactate, highly produced by both stromal and tumor compartments [3,188], contributes to the generation of an immunosuppressive microenvironment in melanoma [67]. Fischer et al. demonstrated that the accumulation of lactate in the melanoma microenvironment inhibits lactate export from cytotoxic T cells through the lactate transporter MCT-1. Consequently, the intracellular accumulation of lactate impairs cytotoxic T cell metabolism and functions, by blocking their proliferation, cytokine production and cytotoxic activity [68]. Furthermore, extracellular acidification due to lactate accumulation in the melanoma microenvironment is associated with the polarization of tumor-associated macrophages (TAMs) toward the tumor-promoting M2 phenotype [3]. The increase of L-Arginine metabolism observed in melanoma and other human cancers, is involved in tumor progression, associated with neovascolarization and immune suppression induction [189]. In particular, L-arginine depletion in the TME induces T cell suppression facilitating the melanoma escape from immunosurveillance [3,187]. Of note, Ersek et al. showed an increased L-arginase activity in MAFs. MAFs were shown to suppress cytotoxic T cell functions via L-arginine depletion (Figure 3). Furthermore, the selective arginase inhibition blocks MAF-induced TIGIT and BTLA expression on cytotoxic T cells [187]. This experimental evidence is in line with other studies showing that arginase inhibition can increase the efficiency of the PD-1/PD-L1 blockade [190,191]. Therefore, all these studies suggest that MAFs, as melanoma cells, may contribute to the generation of a tolerogenic and immunosuppressive melanoma microenvironment by altering the availability of immune-modulating metabolites, such as glucose, arginine and lactate, in the extracellular environment (Figure 3). However, further studies are needed to better clarify the possible link between MAF metabolism and the generation of an immunosuppressive melanoma microenvironment.

CAF-target therapy could be a novel and promising therapeutic strategy able to improve the anti-tumor immune response and immunosurveillance in melanoma. Ohshio et al. demonstrated that targeting CAF fibrotic activity with the anti-fibrotic agent tranilast, in transplantable tumor models of lymphoma, Lewis lung carcinomas and melanoma, decreases the infiltration of Treg and myeloid-derived suppressor cells (MDSCs), and enhances cytotoxic CD8+ T cell response [192]. As previously discussed, distinct MAF subsets are associated with different MAF capabilities to influence the immune system [106]. In particular, FAP + MAFs were shown to exhibit a potent immunosuppressive and tumor-promoting phenotype [187]. Their depletion, upon vaccination with an adenoviral-vector in mouse melanoma models, decreases the number and function of immunosuppressive cells, such as monocytic and polymorphonuclear MDSCs within the TME [193]. Therefore, targeting MAFs can represent a promising therapeutic strategy able to eliminate successfully the anti-tumor immune response occurring during melanoma progression. However, further studies are necessary to better clarify and identify other molecular mechanisms regulating MAF-immune mediating functions with the final goal to develop the best therapeutic strategy able to destroy the pro-tumor immunity in melanoma.

## 6. MAF-Induced MAPKi Resistance

The role of MAFs in melanoma drug resistance is an emerging area of cancer research. Indeed, several studies reported that MAFs can build a drug-tolerant microenvironment through the production of soluble factors and/or ECM proteins. In particular, Straussman et al. identified HGF as a necessary and sufficient fibroblast-secreted factor for conferring drug resistance phenotype to melanoma cells (Figure 2). CAF-derived HGF activates the receptor MET and its downstream pathways MAPK and PI3K/AKT signalling in melanoma cells and leads to immediate resistance to RAF inhibition. Immunohistochemistry analysis of paraffin sections from melanoma patients shows a positive correlation between stromal HGF, innate resistance to treatment and poor response to MAPKi therapy [194]. Stromal HGF’s role in the induction of therapy resistance in melanoma was also confirmed by the work of Wilson’s group [195]. Secreted frizzled-related protein 2 (sFRP2) is a Wnt-antagonist highly secreted by aged fibroblasts and involved in the development of BRAFi resistance in melanoma cells (Figure 2). SFRP2 decreases β-catenin, microphthalmia-associated transcription factor (MITF) and consequently APE1, which is a key redox effector. Loss of APE1 renders melanoma cells more sensitive to ROS-induced DNA damage and increases resistance to BRAFi treatment [196]. Interestingly, Young et al. demonstrated that inflammatory niches consisting of TAMs and MAFs establish a mutation-independent MAPKi tolerance. In particular, MAPKi can enhance the release of macrophage-derived IL-1β that in turn induces the production of CXCR2 ligands in fibroblasts. Consequently, IL-1β-stimulated MAFs enhance the survival of MAPKi-treated melanoma cells in an ERK-independent manner, via NF-κB activation and bcl2 upregulation (Figure 2) [112]. Hirata et al. demonstrated that after treatment with the BRAFi PLX4720, the MAF-derived matrix abrogates the anti-tumor effects of BRAFi by leading to ERK reactivation and cell survival in melanoma cells via adhesion-dependent β1-integrin-FAK-Src signalling. This protective stromal signalling renders BRAF-mutant melanoma cells resistant to BRAFi treatment [152]. Furthermore, it has been reported that MAF-derived matrix enriched of fibrillar and non-fibrillar collagens promotes the clustering of the collagen receptors DDR1/DDR2 into linear membrane structures in melanoma cells. Interestingly, DDR1/DDR2 clustering on the collagen-rich matrix is increased upon melanoma cell treatment with BRAFi. Therefore, this experimental evidence suggests that the treatment with MAPKi can fuel a self-feeding mechanism involving collagen-bound DDR1/DDR2 signalling, which drives collagen network compaction and drug resistance. In particular, DDR1/DDR2 induces matrix-mediated drug resistance to MAPKi via activation of the pro-survival NIK/IKKα/NFκB2 pathway in melanoma cells. DDR1/DDR2 knockdown or inhibition of their catalytic activity increases BRAFi efficacy [149]. Furthermore, it is noteworthy that MAFs arising from melanoma cells that have acquired a CAF/myofibroblast-like phenotype can generate a collagen- and fibronectin-rich protective niche, that allows melanoma resistance to MAPKi (Figure 2) [108,109,110]. In particular, activation of α5β1 integrin/PI3K/AKT signaling pathway and enhanced expression of the pro-survival myeloid cell leukemia 1 (Mcl-1) protein are associated with therapeutic escape and MAPKi resistance in melanoma [108,110].

## 7. Conclusions

Fibroblasts represent a very heterogeneous and plastic cell population regulating both the homeostasis of connective tissue and the inflammatory process and being involved in pathological conditions [80,105,197,198]. In particular, fibroblast populations are able to shift from an inactivated phenotype of quiescent fibroblasts to an activated phenotype of myofibroblasts or a constitutively activated phenotype of MAFs, depending on environmental modifications and cellular interactions [2,80]. This phenotypic plasticity has a dramatic impact on dermal microenvironment homeostasis, cancer growth and therapeutic outcome. Moreover, in vitro and in vivo studies have pointed out that normal fibroblasts and MAFs can regulate differently melanoma growth and dissemination [22,92]. In particular, normal fibroblasts can reduce melanoma cell in vitro vitality and migratory capacity through paracrine interactions [54], and can hinder in vivo tumor formation at the beginning of CM development [92]. Conversely, MAFs, representing the most abundant stromal cells of the melanoma microenvironment, induce TME remodelling, sustain cancer growth and influence therapeutic outcomes [2]. Therefore, we think that the study and a deepened comprehension of signalling pathways and molecules influencing paracrine interactions between normal fibroblasts, before MAF differentiation, and cancer cells could allow the development of more effective therapeutic strategies in blocking melanoma development. Moreover, the study of pathways and molecules regulating the differentiation of MAFs and their interaction with cancer and stromal cells could be pivotal to counteract the pro-tumorigenic capacity of TME.

## Data Availability

Not applicable.
